# Gene Remodeling in Type 2 Diabetic Cardiomyopathy and Its Phenotypic Rescue with SERCA2a

**DOI:** 10.1371/journal.pone.0006474

**Published:** 2009-07-31

**Authors:** Ioannis Karakikes, Maengjo Kim, Lahouaria Hadri, Susumu Sakata, Yezhou Sun, Weijia Zhang, Elie R. Chemaly, Roger J. Hajjar, Djamel Lebeche

**Affiliations:** 1 Cardiovascular Research Center, Mount Sinai School of Medicine, New York, New York, United States of America; 2 Bioinformatics Laboratory of Personalized Medicine Research Program, Mount Sinai School of Medicine, New York, New York, United States of America; 3 Department of Physiology II, Nara Medical University, Kashihara, Nara, Japan; Harvard Medical School, United States of America

## Abstract

**Background/Aim:**

Diabetes-associated myocardial dysfunction results in altered gene expression in the heart. We aimed to investigate the changes in gene expression profiles accompanying diabetes-induced cardiomyopathy and its phenotypic rescue by restoration of SERCA2a expression.

**Methods/Results:**

Using the Otsuka Long-Evans Tokushima Fatty rat model of type 2 diabetes and the Agilent rat microarray chip, we analyzed gene expression by comparing differential transcriptional changes in age-matched control versus diabetic hearts and diabetic hearts that received gene transfer of SERCA2a. Microarray expression profiles of selected genes were verified with real-time qPCR and immunoblotting. Our analysis indicates that diabetic cardiomyopathy is associated with a downregulation of transcripts. Diabetic cardiomyopathic hearts have reduced levels of SERCA2a. SERCA2a gene transfer in these hearts reduced diabetes-associated hypertrophy, and differentially modulated the expression of 76 genes and reversed the transcriptional profile induced by diabetes. In isolated cardiomyocytes *in vitro*, SERCA2a overexpression significantly modified the expression of a number of transcripts known to be involved in insulin signaling, glucose metabolism and cardiac remodeling.

**Conclusion:**

This investigation provided insight into the pathophysiology of cardiac remodeling and the potential role of SERCA2a normalization in multiple pathways in diabetic cardiomyopathy.

## Introduction

Cardiovascular disease is the leading cause of death in diabetic patients [1]. Both clinical and experimental studies have shown that diabetes-induced cardiomyopathy is an important contributing factor of heart failure in diabetic patients independent of atherosclerosis, hypertension, and other complications [2]–[4]. Several metabolic complications, accompanied by insulin deficiency or impaired insulin responsiveness and calcium handling abnormalities, are common to both types 1 and 2 diabetes [5], [6]. The genetic and cellular mechanisms underlying the pathophysiology of diabetes-induced cardiomyopathy have been explored extensively in animal models. These animals have characteristic abnormalities that include altered functional activity of ion channels and pumps and changes in gene expression of regulatory and modulatory proteins of excitation-contraction coupling [7]–[12].

Previous studies show the impairment of SR function in diabetic cardiomyopathy is caused by reduced activity of the SR calcium pump (SERCA2a) due primarily to a decrease in SERCA2a expression [10], [13]–[15] and a 2–4 fold increase in expression of phospholamban (PLB) [10]. With a decrease in SERCA2a expression and an increase in PLB expression, the SERCA2a/PLB ratio is significantly decreased, leading to a slower relaxation. In neonatal rat myocytes *in vitro*, overexpression of SERCA2a largely rescued the phenotype created by increasing the SERCA2a/PLB ratio [16]. In human cardiomyocytes isolated from the left ventricle of patients with end-stage heart failure, gene transfer of SERCA2a resulted in an increase in both protein expression and pump activity, and induced a faster contraction velocity and enhanced relaxation velocity, thereby restoring these parameters to levels observed in nonfailing hearts [17]. In a rat model of pressure-overload hypertrophy in transition to failure, where SERCA2a protein levels and activity are decreased and severe contractile dysfunction is present, overexpression of SERCA2a by gene transfer *in vivo* restored both systolic and diastolic function to normal levels [18], [19]. Normalization of calcium handling also improved survival, normalized altered myocardial metabolism and intracellular signaling pathways [18], and abrogated ventricular arrhythmias [20]. Transgenic diabetic mice overexpressing SERCA2a were also found to have improved cardiac contractile performance and Ca^2+^ handling compared to diabetic wild type mice [21]. Recently, we showed in a type 2 diabetic model that diabetes is associated with cardiac energy wasting with regard to Ca^2+^ regulation. This energy mishandling is demonstrated by the high myocardial oxygen consumption to support left ventricular contractility, which contributes to the contractile dysfunction observed in diabetic cardiomyopathy [22]. Myocardial gene transfer of SERCA2a in these diabetic subjects restored the oxygen cost of left ventricular contractility, as well as contractile dysfunction, to non-diabetic levels [22]. Therefore, SERCA2a appears to improve not only mechanical but also energetic function of the diabetic myocardium by transforming inefficient energy utilization into a more efficient state, in addition to restoring diastolic and systolic function to normal.

Collectively, the positive effects produced by SERCA2a correlate with transcriptional changes that may provide important clues as to the critical pathways involved in cardiac function. In this study we aimed to: 1- explore the changes in gene expression profiles accompanying type 2 diabetes-induced cardiomyopathy and to identify molecular and cellular signaling pathways and genes that may contribute to cardiac remodeling as a result of the disease; and 2- examine the transcriptional changes induced by SERCA2a gene transfer into diabetic hearts and to differentiate between SERCA2a-regulated and diabetes-regulated genes. Functional analysis of the obtained transcriptional profiles indicated that SERCA2a restoration is associated with changes in cellular energetics and metabolism, in calcium handling and in intracellular signaling pathways.

## Materials and Methods

### Construction of recombinant adenoviruses

For the generation of E1 deleted SERCA2a and β-galactosidase (β-Gal) adenoviruses we used the pAdEasy-1 adenoviral plasmid and the pAdTRACK shuttle vector, containing green fluorescent protein (GFP) under the control of the CMV promoter. The Adenovirus β- galactosidase (Ad.β-Gal) was used as a control. The titers of stocks used in these studies measured by plaque assays were 5.9×10^10^ pfu/ml (Ad.SERCA2a) and 4.5×10^10^ pfu/ml (Ad.β-Gal), with a particle/pfu ratio of 40∶1. Wild-type adenovirus contamination was excluded by the absence of PCR-detectable early region 1 (E1) sequences.

### Animals

Five-week-old male diabetic Otsuka Long-Evans Tokushima Fatty (OLETF) and normal male Long-Evans Tokushima Otsuka (LETO) rats were obtained from Tokushima Research Institute, Otsuka Pharmaceutical Company (Tokushima, Japan). The OLETF is an established model of spontaneous non-insulin-dependent type 2 diabetes mellitus (DM) that manifests stable clinical and pathological features that resemble human type 2 DM. The model is characterized by hyperinsulinemia from 8 weeks of age, insulin resistance of the peripheral tissues from 12 weeks of age, late onset of hyperglycemia after 20 weeks of age, and diagnosable DM by oral glucose tolerance test (OGTT) from 25 weeks of age [23], [24]. Furthermore, the OLETF rats (60–66 weeks of age) develop significant slowing of isovolumic LV relaxation rate with depressed expression of cardiac SERCA2a protein [25].

### Adenoviral delivery protocol

Sixty to sixty five week-old OLETF rats, with clear systolic and diastolic dysfunction, were randomized into 3 groups: diabetic group with no gene transfer (DM), diabetic group with adenoviral SERCA2a gene transfer (DM+Ad.SERCA2a), and diabetic group with adenoviral β-galactosidase gene transfer (DM+Ad.β-Gal). LETO rats served as non-diabetic control animals. The adenoviral delivery system has been described previously [26]. Four to six days after adenoviral transduction, the hearts were harvested, separated into right or left ventricles, weighed and then frozen in liquid nitrogen and stored at −80°C.

### Preparation of cRNA, direct labeling and oligonucleotide array hybridization

Total RNA (1 µg), isolated from LETO and OLETF hearts transduced with Ad.SERCA2a or Ad.β-Gal, was amplified and the cyanine-3/cyanine-5 labeled CTP was incorporated using T7 RNA polymerase. Equimolar amounts of cRNAs from control (labeled with Cy5) and DM, DM+SERCA2a or DM+β-Gal (labeled with Cy3) hearts were mixed together and were cleaned through QIAquick PCR Purification Kit spin columns (Qiagen, Valencia, CA) to remove unincorporated dye-labeled nucleotides. The concentration and pmol incorporation was calculated after absorbance readings were taken at 260, 280, 550 and 650 nm. To perform reverse labeling, equimolar amounts of oppositely labeled cRNAs from the infected and uninfected hearts were mixed and hybridized to a separate microarray slide. The slides were hybridized, washed and scanned according to Agilent recommendations and settings.

### Scanning and Feature Extraction of arrays

Arrays were scanned with Agilent's G2565AA Microarray Scanner System and analyzed using Agilent Feature extraction software (Agilent Technologies), which calculates log ratios and *P* values for valid features on each array and provides a confidence measure of gene differential expression by performing outlier removal, background subtraction, and dye normalization for each feature. The software filters features that are not positive and significant with respect to background or features that are saturated. It then fits a normalization curve across the array using the locally weighted linear regression curve fit (LOWESS) algorithm to detect and correct dye bias.

### Analysis of microarray data

Differential expression values are presented as ratio of intensities between diabetic or diabetic-treated (with SERCA2a or β-Gal) and control samples. Expression data were omitted in the regions where no signal was present or if the signal was just above local background or derived from <40% of the area of the printed spot. After normalization of the intensities, the data were filtered to exclude spots with intensities less than twice the background (150 pixels) in either channel and finally, only spots with a normalized ratio greater than 1.5 were considered, since a minimum of 1.4-fold change in differential expression can be accurately detected [27]. However, only data with 2 fold-change or higher cutoff values are presented in these studies. Using the processed data we then performed in depth data analysis with the Genespring software (Agilent Technologies). The significantly regulated genes were grouped into functional categories based on annotation by Gene Ontology, pathway (KEGG and Ingenuity) analysis and PIR keywords using Genespring and DAVID version 2007.

### Extraction of DM and SERCA2a affected genes

We compared the microarray data among the various groups: control non-diabetic vs. diabetic (DM); control non-diabetic vs. DM+SERCA2a; control non-diabetic vs. DM+β-Gal; DM vs. DM+SERCA2a; DM vs. DM+β-Gal. First, a list of genes (number = 838) differentially expressed (2-fold or more change in expression levels) between DM and control samples was generated. Within this list, genes differentially expressed (2-fold or more) between DM+SERCA2a and DM samples were selected and a second list of genes was generated using the Venn-diagram approach. From the latter list, genes that had a more than 2-fold change in expression level between the DM samples and the DM+β-Gal group were excluded since they likely represent the effect of the viral gene transfer. In summary, we first identified the genes affected by the DM, and among those genes, the ones affected by SERCA2a gene therapy and we subtracted the effect of β-Gal from that of SERCA2a to extract SERCA2a specifically-regulated genes. Differences in gene expression between groups were evaluated by using the *t* test with unequal variances. Annotations were compiled by using Genespring and Ingenuity softwares.

### qRT-PCR analysis and western blotting

Expression of selected genes was determined by using two-step quantitative real-time PCR. Total RNA (2 µg), from control and DM hearts, was reverse transcribed using the High Capacity cDNA Reverse Transcription kit (Applied Biosystems (ABI), Foster City, CA) according to the manufacturer's protocol and qPCR was performed with Power SYBR green PCR Master Mix on an ABI Prism 7500 Real Time PCR System. Multiple transcripts were analyzed simultaneously for 40 cycles using an optimized qRT-PCR thermal profile. Data Analysis was performed using Real-Time SDS software (ABI). For each set of primers, a no template control and a no reverse amplification control were included. Post-amplification dissociation curves were performed to verify the presence of a single amplification product in the absence of DNA contamination. Fold changes in gene expression were determined using the ΔΔCt method with normalization to 18S rRNA endogenous control.

Following qRT-PCR analysis, corresponding protein changes of some putative mRNA changes were analyzed by immunoblotting using standard protocol. Films from at least three independent experiments were scanned and densities of the immunoreactive bands were evaluated using NIH Image software.

### Protein synthesis rate measurements

Protein synthesis rates in neonatal cardiomyocytes were determined using [^3^H]-Leucine incorporation as described previously [28]. [^3^H]-Leucine incorporation was measured by scintillation counting (MicroBeta Trilux, PerkinElmer). [^3^H]-Leucine uptakes were measured by stimulating the myocytes with Ad.SERCA2a or Ad.β-gal (MOI 50) in the presence or absence of endothelin-1 (100 nM).

### Statistical analysis

All values were calculated as mean±SD. Data were compared by two-tailed Student's *t* test. The null hypothesis was rejected for *P*<0.05.

## Results

### Characterization of animals and ventricular function

**Figure 1:**
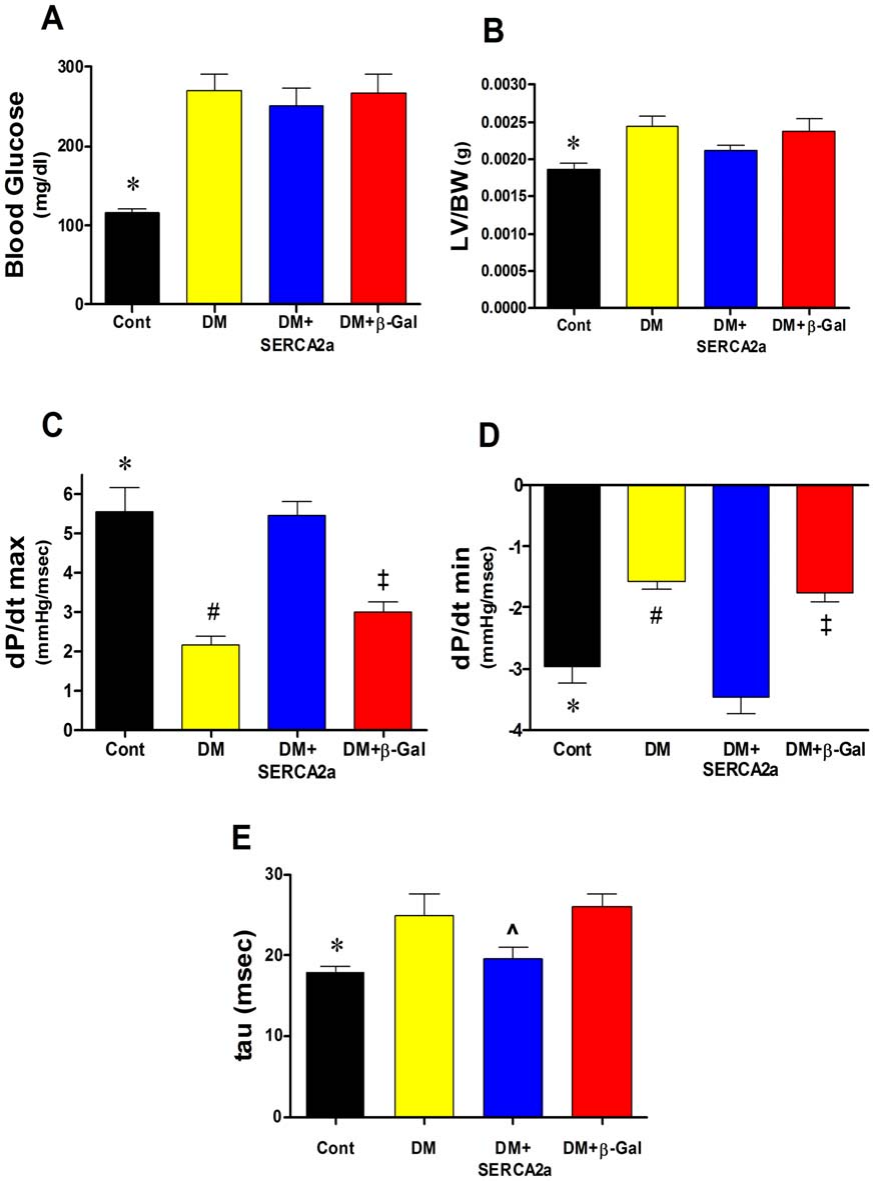
Cardiac structural and functional parameters in the different experimental animal groups. (A) Blood glucose levels. Diabetes (DM) induced a sharp increase in glucose levels which remained high even after viral infection with SERCA2a and β-gal transgenes. (B) LV/BW ratio calculated based on the LV weight and the final weight of the animal (prior to death). Changes in contractility (dP/dt max) (C), in relaxation (dP/dt min) (D) and in tau (E) in the different groups. All data shown as means±SD. LV, left ventricle; BW, body weight. ^*^ *P*<0.05 Control vs. DM; ^#^ *P*<0.01 DM vs. DM+SERCA; ^‡^ *P*<0.05 DM+β-Gal vs. DM+SERCA; ^ *P* = 0.04 DM+SERCA vs. DM+β-Gal.

The Otsuka Long–Evans Tokushima Fatty (OLETF) rat is an established model of congenital Diabetes Mellitus (DM) which shows left ventricular (LV) diastolic dysfunction and slowing of isovolumic LV relaxation rate associated with abnormal calcium handling and depressed SERCA2a protein expression [23], [25]. The development of diabetes was confirmed by a marked increase in blood glucose levels in all DM rats, measured after 5–6 hours of fasting, compared to non-DM control animals (Fig. 1A). The body weights (BW) were not statistically different in all groups (not shown). The data in figure 1B also show a significant increase in LV/BW ratio in all DM rats (2.45×10^−3^ g±0.1, n = 8) compared with normal rats (1.87×10^−3^ g±0.08, n = 9, P<0.003), an indication of cardiac hypertrophy. The mean LV/BW in DM+SERCA2a group significantly decreased compared to DM group (2.13×10^−3^ g±0.07, n = 4, P<0.04) but did not reach the control levels (Fig. 1B), which may indicate that SERCA2a gene transfer into diabetic hearts reverses the associated hypertrophy.

Compared to controls, the DM rats showed severe left ventricular (LV) diastolic and systolic dysfunction. The maximal rate of LV pressure rise (dP/dt max) and LV pressure fall (dP/dt min) were significantly decreased in DM group (Fig. 1C & D, respectively). This observed cardiac dysfunction is likely due to decreased levels of SECRA2a expression [22] since SERCA2a gene transfer into DM hearts dramatically reversed these parameters (dP/dt max 5.46±0.59 vs. 2.15±0.55 mmHg/msec, P<0.05; dP/dt min −3.46±0.48 vs. −1.58±0.28 mmHg/msec, n = 6, P<0.05) (Fig. 1C & D). In addition, SERCA2a transduction significantly improved the time course of relaxation (tau) compared to DM hearts (19.6±2.4 vs 25.0±5.8 msec, P<0.05) (Fig. 1E). These data indicate that SERCA2a gene transfer into DM hearts considerably improved myocardial performance. Of note, these SERCA2a-mediated improvements were not the result of viral infection since control virus (β-Gal) did not affect *in vivo* cardiac function (Fig. 1C, D & E).

### Microarray data analysis

**Figure 2:**
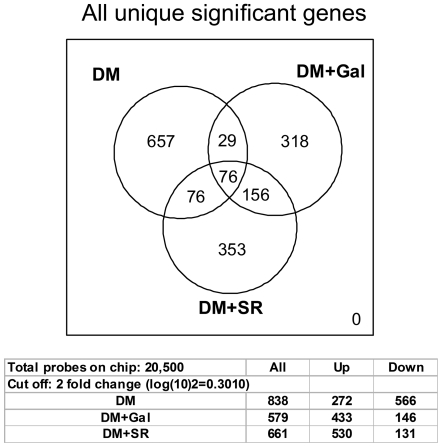
Venn diagram representing the sets of transcripts comparison. Seventy six genes were differentially regulated by DM and SERCA2a. Also shown are the total numbers of genes differentially modified in the 3 experimental conditions. DM, hearts with diabetes mellitus; DM+SR, diabetic hearts infected with Ad.SERCA2a; DM+Gal, diabetic hearts infected with Ad.β-Gal.

We used Agilent's 60-mer oligonucleotide two-color dye assay and quantitative real-time PCR to identify differentially expressed genes in type 2 diabetic failing hearts and their phenotypic rescue by SERCA2a restoration. Raw data output was imported into Genespring GX7.3 (Agilent Technology) and normalized by setting values below 0.01 to 0.01, normalizing each chip to the 50^th^ percentile of all measurements in that sample (per chip normalization), and normalizing each gene to the median measurement for that gene across all samples (per gene normalization). To focus on genes with reliable changes in expression, we filtered the normalized data for signal intensity with a ratio of ≥2 for up regulation and a ratio of ≤0.5 for down-regulation. We compared the microarray datasets among the various groups: DM; DM+SERCA2a and DM+β-Gal (Fig. 2). The diabetic subjects were compared with control subjects. Ad.β-Gal was used as a viral control vehicle and its effect removed from that of Ad.SERCA2a to extract SERCA2a specifically-regulated genes (Fig. 2).

The results reveal a significant divergence in gene expression among the DM and the two adenoviral treatments (Fig. 2), with a total of 2078 transcripts being differentially expressed. The data from the 3 comparisons (DM vs. Cont = 838 genes; DM vs. DM+SERCA2a = 661 genes; DM vs. DM+β-Gal = 579 genes) revealed few commonly regulated genes (Fig. 2). Of particular interest to our laboratory is the set of 76 genes that were co-regulated by both DM and SERCA2a overexpression. This will help us explore the nature of genes that are affected by diabetes, and their nature following phenotypic rescue by SERCA2a restoration.

### Diabetes-induced transcriptional profile

Of the more than 20,000 genes represented on the array, 838 transcripts were differentially expressed between control and diabetic hearts (Fig. 2). Of 838 genes, 272 were up-regulated and 566 were down-regulated, an indication that diabetes is associated with a net suppression of gene expression (Fig. 2). A partial list of these transcripts ranked according to greatest fold change in expression is shown in Table 1. Among the genes that were significantly up-regulated in diabetes were Retnla, Gjb2, Itga, Slit and Dusp6, and among the genes that were down-regulated were Krueppel-like factor 8, IGFBP, Ngfg, Foxa3, Kcnc2 and Dlp8. Gene Ontology (GO) analysis confirmed these findings with processes such as cell-cell signaling, immune response, development, intracellular signaling, proliferation and transcription. These processes are overrepresented as some of the most significant terms for the diabetes class with *P* values<0.05 (see below).

**Table 1:** Top 25 genes differentially regulated by diabetes.

Up-regulated genes				
Systematic Name	Gene Name	Description	Fold change	*P* value
NM_053333	**Retnla**	Resistin-like alpha	**54.03**	0.0487
NM_001004099	**Gjb2**	Gap junction membrane channel protein beta 2	**9.2**	0.011
NM_017327	**Gnao**	Guanine nucleotide binding protein, alpha o	**5.75**	0.022
XM_220398	**LOC303113**	Similar to FLJ00195 protein	**5.21**	3.00E-04
NM_030994	**Itga1**	Integrin alpha 1	**4.69**	0.109
BC088446	**Nostrin**	Dab2-interacting protein 2/nitric oxide synthase trafficker	**4.37**	0.0289
NM_016994	**C3**	Complement component 3	**4.03**	0.027
NM_040669	**Hps1**	Hermansky-Pudlak syndrome 1 homolog (human)	**3.99**	0.0237
NM_183330	**Ctsz**	Cathepsin Y	**3.92**	0.0455
NM_012569	**Gls**	Glutaminase	**3.89**	0.05
NM_052809	**Cdo1**	Cytosolic cysteine dioxygenase 1	**3.88**	0.0346
XM_235156	**LOC314843**	Similar to vascular endothelial protein tyrosine phosphatase	**3.75**	0.0491
NM_022698	**Bad**	Bcl2-associated death promoter	**3.73**	0.0495
NM_147207	**Vof16**	Ischemia related factor vof-16	**3.69**	0.0042
XM_238467	**isg12(b)**	Hypothetical LOC299269	**3.65**	0.001
NM_031321	**Slit3**	Slit homolog 3 (Drosophila)	**3.63**	0.0147
NM_001002805	**C4-2**	Complement component 4, gene 2	**3.63**	0.0149
NM_031531	**Spin2c**	Serine protease inhibitor	**3.39**	0.0473
NM_181384	**Tnfsf9**	Tumor necrosis factor (ligand) superfamily, member 9	**3.29**	0.0499
NM_053667	**Lepre1**	Leprecan	**3.26**	0.0237
NM_030829	**Gprk5**	G protein-coupled receptor kinase 5	**3.25**	0.0305
NM_001004253	**Syap1**	SYAP1 protein	**3.19**	4.00E-04
U42627	**Dusp6**	Dual-specificity protein tyrosine phosphatase (rVH6)	**3.18**	0.0466
NM_138882	**Pspla1**	Phosphatidylserine-specific phospholipase A1	**3.18**	0.0428
NM_021760	**Col5a3**	Collagen, type V, alpha 3	**3.16**	0.0259

Down-regulated genes				
Systematic Name	Gene Name	Description	Fold change	*P* value
XM_228805	**LOC302582**	Similar to Krueppel-like factor 8	**0.258**	0.0064
NM_012595	**Ldhb**	Lactate dehydrogenase B	**0.254**	0.0017
XM_214266	**Tnnc1**	Similar to troponin C, cardiac muscle -	**0.251**	0.0176
NM_053329	**Igfals**	Insulin-like growth factor binding protein, acid labile subunit	**0.241**	0.113
NM_017239	**Myh6**	Myosin heavy chain, polypeptide 6	**0.227**	0.0191
S74398	**S74398**	L-type Ca2+channel alpha 1 subunit	**0.215**	0.0185
X04240	**Mor1**	Mitochondrial malate dehydrogenase	**0.214**	0.0243
NM_031523	**Ngfg**	Nerve growth factor, gamma	**0.213**	0.0460
NM_017077	**Foxa3**	Forkhead box A3	**0.211**	0.0258
NM_019207	**Neurod3**	Neurogenic differentiation 3	**0.211**	0.0259
NM_022217	**Amph1**	Amphiphysin	**0.21**	0.0261
NM_022850	**Dpp6**	Dipeptidylpeptidase 6	**0.209**	0.0263
NM_053867	**Tpt1**	Tumor protein, translationally-controlled 1	**0.208**	0.0292
M59313	**Kcnc2**	Potassium channel Kv3.2c	**0.207**	0.03
NM_021841	**Gabra6**	Gamma-aminobutyric acid A receptor, alpha 6	**0.206**	0.0313
XM_238787	**LOC308516**	Similar to Rhophilin-2	**0.204**	0.0338
XM_236415	**LOC315824**	Similar to Bone morphogenetic protein 5 precursor	**0.203**	0.0327
XM_228058	**Dlp8**	Dynein-like protein 8	**0.197**	0.033
XM_221672	**Tiam1**	Similar to invasion inducing protein	**0.181**	0.0466
NM_031044	**Hnmt**	Histamine N-methyltransferase	**0.174**	0.0308
NM_054000	**Kcnb2**	Potassium voltage gated channel, Shab-related subfamily, member 2	**0.173**	0.0357
XM_215287	**Kif11**	Kinesin-like 1	**0.152**	0.0369
NM_053364	**Plfr**	Proliferin-related protein	**0.149**	0.0367
AF169825	**Chd8**	β-Catenin binding protein/chromodomain helicase DNA binding protein 8	**0.134**	0.0423
NM_013191	**S100b**	S100 protein, beta polypeptide	**0.117**	0.0372

### Gene Ontology analysis and pathway identification of diabetes-induced transcripts

**Figure 3:**
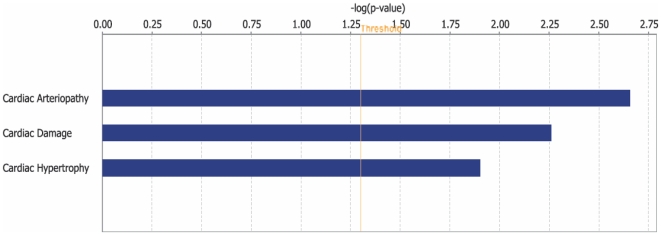
Diabetes cardiotoxicity function. Ingenuity analysis of the DM-induced transcriptional profile indicated that diabetes has markedly impacted cardiac function. *P* value is the likelihood that a given pathway is associated with a gene set by random chance.

Using the Gene Ontology (GO) database, we categorized the 838 differentially expressed genes into known or probable functional categories (Table 2). The GO Biological Process category with the highest number of differentially expressed genes was development (77 genes, 15.78%), consistent with an active diabetes-induced cardiac remodeling process. Genes like fibroblast growth factor receptor 1, syntrophin, transcription factor EC, fibronectin 1 and slit homolog 3 were classified in this group. Genes involved in response to stress, immune response and response to wounding were also significantly represented in GO Biological Process categories, which is consistent with the finding that diabetic cardiomyopathy is associated with increased stress levels leading to immune dysregulation that negatively impact the heart. Genes included in this group were those encoding for chemokines and those encoding members of the complement system. The cellular signaling category contained genes like protein Kinase D2, frizzled homolog 2, dual specificity phosphatase 6, mitogen activated protein kinase kinase kinase 10, adrenergic receptor alpha 2a, and insulin-like growth factor 1, amongst others. GO Molecular Function analysis showed that cell-to-cell signaling and interaction, metabolism and energy pathways were altered, consistent with the observation that diabetes is closely associated with metabolic dysregulation [29]. In addition, Ingenuity Pathway Analysis showed that the top cardiotoxicity functions induced by diabetes are cardiac arteriopathy, cardiac damage (injury to myocardium) and cardiac hypertrophy (Fig. 3), which may explain the link between diabetes and cardiac hypertrophy and heart failure.

**Table 2:** Functional categories associated with diabetic cardiomyopathy.

Term	Count	%	*P* Value
**Biological Process**			
development	77	15.78%	0.0064
response to stress	46	9.43%	8.55E-04
defense response	45	9.22%	1.58E-05
cell adhesion	36	7.38%	3.87E-07
cell differentiation	36	7.38%	0.0081
intracellular signaling cascade	34	6.97%	0.0182
cell-cell signaling	33	6.76%	3.23E-04
response to external stimulus	30	6.15%	1.14E-04
response to wounding	29	5.94%	4.34E-06
cell proliferation	29	5.94%	0.0047
cell motility	19	3.89%	0.0067
cell migration	15	3.07%	0.0053
cell activation	12	2.46%	0.0074
muscle contraction	11	2.25%	0.0045
induction of apoptosis	11	2.25%	0.0202
nucleotide metabolism	11	2.25%	0.0310
ion homeostasis	10	2.05%	0.0445
transmembrane receptor protein tyrosine kinase signaling pathway	9	1.84%	0.05
**Cellular Components**			
extracellular region	88	18.03%	4.42E-06
plasma membrane	70	14.34%	0.0019
intrinsic to plasma membrane	43	8.81%	0.0061
integral to plasma membrane	42	8.61%	0.0082
extracellular matrix	13	2.66%	0.0169
sarcomere	6	1.23%	0.0127
myofibril	6	1.23%	0.0190
contractile fiber	6	1.23%	0.0347
**Molecular Function**			
protein binding	129	26.43%	0.0020
ion binding	60	12.30%	0.0417
calcium ion binding	35	7.17%	2.03E-06
receptor binding	26	5.33%	0.0221
electrochemical potential-driven transporter activity	12	2.46%	0.0100
cytokine activity	10	2.05%	0.05
lipid binding	10	2.05%	0.0430
oxidoreductase activity, acting on CH-OH group of donors	8	1.64%	0.0329
hormone activity	8	1.64%	0.0373
phospholipid binding	7	1.43%	0.0354
protein tyrosine phosphatase activity	6	1.23%	0.0485
**KEGG Pathway**			
RNO01430:CELL COMMUNICATION	9	1.84%	0.05
RNO04512:ECM-RECEPTOR INTERACTION	8	1.64%	0.0183
RNO04610:COMPLEMENT & COAGULATION CASCADES	7	1.43%	0.0165
RNO00630:GLYOXYLATE AND DICARBOXYLATE METABOLISM	3	0.61%	0.0396
**SP_PIR_Keywords**			
glycoprotein	75	15.37%	1.33E-06
transmembrane	75	15.37%	1.93E-05
membrane	75	15.37%	4.11E-05
signal	69	14.14%	1.21E-08
receptor	37	7.58%	0.0203
hydrolase	31	6.35%	0.02645
calcium	23	4.71%	4.92E-04
transducer	17	3.48%	0.0458
cell adhesion	16	3.28%	6.13E-06
g-protein coupled receptor	16	3.28%	0.0393
phosphoprotein	16	3.28%	0.0457
acetylation	13	2.66%	0.0183
developmental protein	13	2.66%	0.0209
egf-like domain	9	1.84%	0.0062
cytokine	7	1.43%	0.0183
inflammatory response	6	1.23%	0.0012
hormone	6	1.23%	0.0380
sulfation	5	1.02%	0.0021
muscle protein	5	1.02%	0.0306

Categories ascribed to genes were determined from the Gene Ontology (GO) listings found in Genespring, DAVID and Ingenuity. Count represents the number of genes associated with a specific term. GO annotation may classify genes more than once in any given category.

### SERCA2a-induced transcriptional profile in DM hearts

We were particularly interested to discover changes in LV gene expression profile following rescue of diabetic cardiomyopathy via cardiac overexpression of SERCA2a. SERCA2a gene transfer in diabetic (DM) hearts differentially induced the expression of 76 genes and appears, in general, to reverse the transcriptional profile induced by diabetes (Fig. 2). Out of the 76 SERCA2a-targeted genes in DM, 43 are upregulated and 33 are down-regulated. A list of the most differentially regulated genes is presented in Table 3.

**Table 3:** Selected list of genes differentially regulated by SERCA2a (2-fold change).

Up-regulated genes				
Gene Name	Fold Change	Common	Genbank	Functional Classification
A_43_P15881	24.09	Olfactory receptor gene Olr1482	NM_001000026	Signal transduction
A_43_P21854	16.51	Unknown		
A_43_P18139	11.96	Myeloid/lymphoid or mixed-lineage leukemia 2	XM_343326	Cell growth and/or Maintenance
A_43_P16951	9.67	Unknown		
A_42_P758644	8.322	HUMCOLVIIA type VII collagen		
A_43_P15539	7.026	Interleukin 8 receptor, alpha	NM_019310	Cell-cell Signaling
A_42_P692126	6.569	Secretogranin 2	NM_022669	Calcium ion binding/chemo-attractant activity/Cytokine activity
A_43_P18005	5.864	Unknown		
A_43_P19121	5.674	Similar to WD repeat membrane protein	XM_223412;	Intracellular protein transport
A_43_P14794	5.448	Unknown		
A_43_P14388	4.815	Unknown		
A_42_P456940	4.805	Unknown		
A_43_P15963	4.708	Synaptotagmin X	NM_031666	Calcium ion binding/transporter activity
A_43_P21893	4.557	Cadherin 16 (predicted)	NM_001012055	Cell adhesion/Calcium ion binding
A_43_P21849	3.781	Similar to ovomacroglobulin, ovostatin	XM_342764	Metabolism (carbohydrates)
A_43_P19860	3.224	Unknown		
A_43_P18464	3.202	Similar to KIAA0774 protein	XM_221871	Unknown
A_43_P22576	3.051	Angiotensin/vasopressin receptor	XM_577848	Signal transduction
A_43_P16129	3.02	Secreted frizzled-related protein 4	AF220608;	Signal transduction/Development/Metabolism
A_43_P21374	2.77	McKusick-Kaufman syndrome protein	NM_001008353	Development/Protein folding
A_43_P15978	2.53	Fibroblast growth factor 4	NM_053809	Cell-cell signaling/Growth/Gene expression
A_43_P22200	2.251	Similar to CG2747-PA	XM_343061	
A_43_P15022	2.249	Phosphoinositide-3-kinase adaptor protein 1	XM_220008	Kinase activity
A_42_P668600	2.203	Neprilysin-like peptidase gamma		Hydrolase/Peptidase activity
A_43_P14187	2.103	Calcium channel, voltage-dependent, β 3 subunit	NM_012828	Ion transport activity
A_43_P15267	2.074	Murine thymoma viral (v-akt) oncogene homolog 2 (akt2)	NM_017093	Signal transduction

Down-regulated genes				
Gene Name	Fold Change	Common	Genbank	Functional Classification
A_43_P12399	0.476	Huntingtin-associated protein 1	U38373	Cytoskeleton organization/Transport
A_43_P22229	0.362	Similar to RIKEN cDNA D430039N05 gene	XM_575160	
A_43_P15400	0.337	Breast cancer 1	NM_012514	Apoptosis/Transcription/Metabolism
A_43_P20674	0.308	Similar to RIKEN cDNA 6030404E16 (predicted)	XM_343366	
A_43_P22798	0.308	**Unknown**		
A_43_P11676	0.254	Trefoil factor 3	NM_013042	Signal transduction
A_43_P19573	0.117	Similar to 2810453L12Rik protein (predicted)	XM_227619	
A_42_P677354	0.0707	Sodium channel, voltage-gated, type 8, alpha polypeptide	NM_019266	Transport activity
A_43_P16076	0.0707	**Unknown**		
A_43_P11351	0.0707	**Unknown**		
A_42_P774782	0.0707	Potassium inwardly-rectifying channel, subfamily J, member 13	NM_053608	Ion channel activity
A_43_P20000	0.0388	Dermatan sulphate proteoglycan 3 (predicted)	XM_235083	Metabolism
A_43_P22206	0.0342	Chemokine (C-C motif) ligand 9 (predicted)	NM_001012357	Cell-cell communication
A_43_P15454	0.0297	Neuromedin	NM_022239	Regulation of gene expression
A_43_P11807	0.0209	Forkhead box A3	NM_017077	Regulation of gene expression
A_42_P833800	0.0123	**Unknown**		
A_43_P12641	0.00864	Claudin 1	NM_031699	Structural molecule activity
A_43_P16113	0.00552	Protocadherin alpha 3	NM_053941	Cell adhesion/Calcium ion binding
A_43_P19694	0.00432	Phosphatidylinositol 3-kinase, catalytic, alpha polypeptide	XM_574916	Protein kinase activity
A_43_P11989	0.00393	Gap junction membrane channel protein beta 3	NM_019240	Cell communication/Transport
A_43_P19821	0.000917	Similar to calmegin (predicted)	XM_222484	Calcium binding/Chaperone activity

### Gene Ontology and Cluster analysis of SERCA2a-induced transcripts

The majority of the genes modulated by SERCA2a appear to be involved in signaling mechanisms. Gene Ontology analysis confirmed this finding with processes such as “cell-cell signaling” and “signal transduction” mechanism (both overrepresented in the top genes with *P* values<0.001) as some of the most significant molecular functions (not shown). Metabolism and calcium signaling are also significantly represented. KEGG analysis showed that SERCA2a-induced transcriptional profile is associated with significant alterations in the MAPK signaling pathway, consistent with gene ontology data. Likewise, the analysis against the Ingenuity database showed that IL-6 signaling, TGF-β and PPAR signaling, and PI3K/Akt signaling were the most significantly overrepresented.

K-means clustering analysis classified the SERCA2a-regulated genes into 4 clusters: genes that were up-regulated (set 1∶10 genes) or down-regulated (set 2∶31 genes) by diabetes and reverted back toward normal expression levels by SERCA2a, and genes that were down-regulated by diabetes but up-regulated (set 3∶12 genes) or further down-regulated (set 4∶23 genes) by SERCA2a overexpression.

### Validation of microarray data following SERCA2a restoration in diabetic hearts

**Figure 4:**
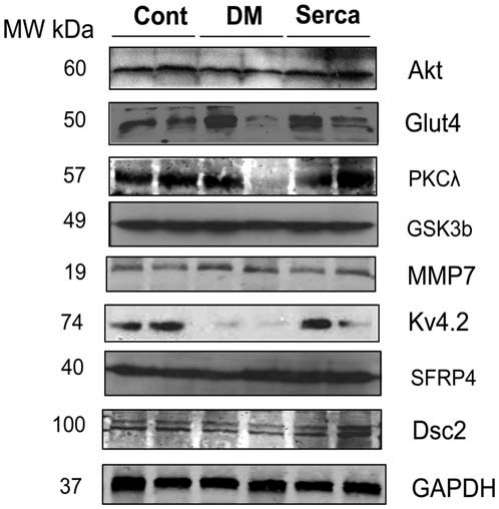
Validation of microarray data by Immunoblotting. Immunoblotting analysis of selected proteins was performed on samples from control, DM and Serca2a-transduced hearts. A representative blot from 2–3 experiments is shown. sFRP4, secreted frizzled-related protein 4; DSC2, desmocollin 2. GAPDH is also shown to verify protein loading. Cont, control non-diabetic; DM, hearts with diabetes mellitus; Serca2a, diabetic hearts infected with Ad.Serca2a; MW, molecular weight.

qRT-PCR was used to verify the oligonucleotide microarray expression data. The differential expressions of 19 randomly selected genes identified by microarray were validated. Relative transcript levels were determined in control non-diabetic and diabetic hearts compared to diabetic hearts transduced with SERCA2a. qRT-PCR analysis confirmed that the majority of genes were statistically differentially regulated (P<0.05) in DM+SERCA2a samples (Table 4). Although the pattern of fold-change of many of the genes, as determined by qRT-PCR analysis, correlated with fold-change reported by microarray analysis, there were some discrepancies in at least 5 cases. The greatest discrepancy was seen in the expression of ryanodine receptor 2 (RyR2), which was reported as 11.4-fold up-regulated in DM hearts by microarray analysis compared with a modest 1.19-fold by qRT-PCR (Table 4).The second major discrepancy was observed in the expression levels of GLUT4, which was reported as 1.30-fold up-regulated by microarray analysis compared with 0.21-fold down-regulation by qRT-PCR (Table 4) in DM samples. GLUT4 expression is widely reported to be down-regulated in the diabetic heart, consistent with our qRT-PCR data. Immunoblotting of GLUT4 protein expression confirmed this observation (Fig. 4) in deference to the microarray data. The other discrepancy between qRT-PCR and microarray was seen with the relative expression of MMP7, 7.08-fold versus 1.63-fold, respectively (Table 4). FGF4 and Cacnb3, although displayed on the microarray, were not amplified by qRT-PCR. Nevertheless, the qRT-PCR results are in general concordance with the microarray data and serve to verify the results. The variations observed could be due to the differences in the techniques and/or the sequences of oligonucleotide probes used in the two approaches.

**Table 4:** Comparisons between fold-change in gene expression determined by microarray and qRT-PCR methods.

	*MICROARRAY*		*qPCR*	
Gene	DM	DM+SERCA	DM	DM+SERCA
Akt1	0.87	1.43	0.29	0.74
Akt2	1.02	2.1	0.19	0.77
PI3Ka	1.01	1.41	1.30	3.16
GSK-3b	1.08	0.68	1.10	0.76
PKC-λ	1.01	1.65	0.88	1.68
GLUT1	0.94	0.68	1.27	0.61
GLUT4	1.30	1.72	0.21	0.91
CK	0.88	1.41	0.59	2.14
Serca2a	0.79	1.08	0.48	2.21
S100A3	1.80	0.68	2.63	1.15
CaBP1	1.13	0.12	1.96	0.98
RyR2	11.4	0.05	1.19	0.94
Cacna1c	1.05	1.55	0.60	1.12
Cacnb3	0.29	0.62	N/A	N/A
MMP7	1.63	0.13	7.08	0.65
FZD4	2.32	0.43	1.50	0.99
FGF4	0.24	0.62	N/A	N/A
sFRP4	0.26	0.78	1.64	2.94
Retnla	54.03	0.27	23.8	0.95

The fold changes in mRNA levels in diabetes (DM) and DM+SERCA2a samples of selected genes were determined by microarray and RT-qPCR. Transcripts were selected based on their roles in insulin signaling, energy/metabolism, Ca^2+^ handling, structural remodeling and intracellular signaling. CK, creatine kinase; sFRP4, secreted frizzled-related protein 4; Retnla, resistin-like alpha; Cacna1c, calcium channel, voltage-dependent, L type, alpha 1C subunit; Cacnb3, calcium channel, voltage-dependent, beta 3 subunit. N/A, not amplified.

For further secondary validation of the microarray data we carried out western blot analysis for several proteins. Data obtained by immunoblotting for these proteins (Fig. 4) involved in a variety of functions, were qualitatively concordant with that found by the microarrays and qRT-PCR (Table 4) with the exception of GSK3β which appears to be unchanged.

### Effect of SERCA2a on transcript levels in isolated cardiomyocytes in vitro

**Figure 5:**
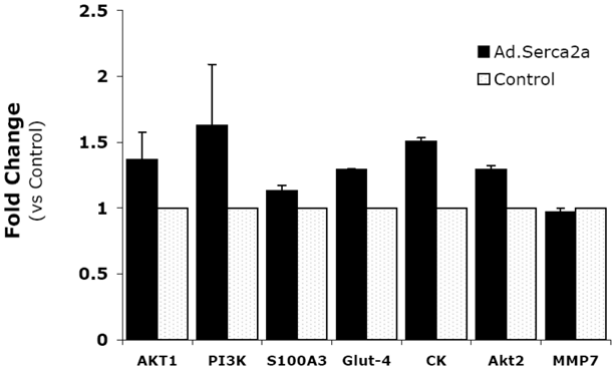
Effects of transient SERCA2a expression on selected transcript levels *in vitro*. Isolated adult ventricular myocytes were infected with Ad.SERCA2a (MOI = 100) for 48 hours, total RNA extracted and relative transcript expression analyzed in real time by qPCR using gene-specific primers. Product specificity was confirmed by post-amplification dissociation curve analysis. Ad.SERCA2a increased the expression of all genes (except MMP-7) thus confirming the effect of SERCA2a on the microarray chip. CK, creatine kinase.

We examined whether the expression of selected transcripts was directly regulated by SERCA2a overexpression, with β-Gal serving as a control, in adult rat ventricular myocytes. Cardiomyocytes were infected with Ad.SERCA2a and Ad. β-Gal for 48 hours and the expression levels of Akt, PI3K, GLUT4, S100a3, MMP7, and CK were determined by qRT-PCR (Fig. 5). The analysis showed that SERCA2a significantly induced the expression of all molecules examined except MMP-7, which is increased in diabetic cardiomyocytes and down-regulated by SERCA2a to normal levels. Normal non-diabetic cardiomyocytes express low levels of MMP-7 which are not changed by SERCA2a.

### Effect of SERCA2a on cardiac hypertrophy

**Figure 6:**
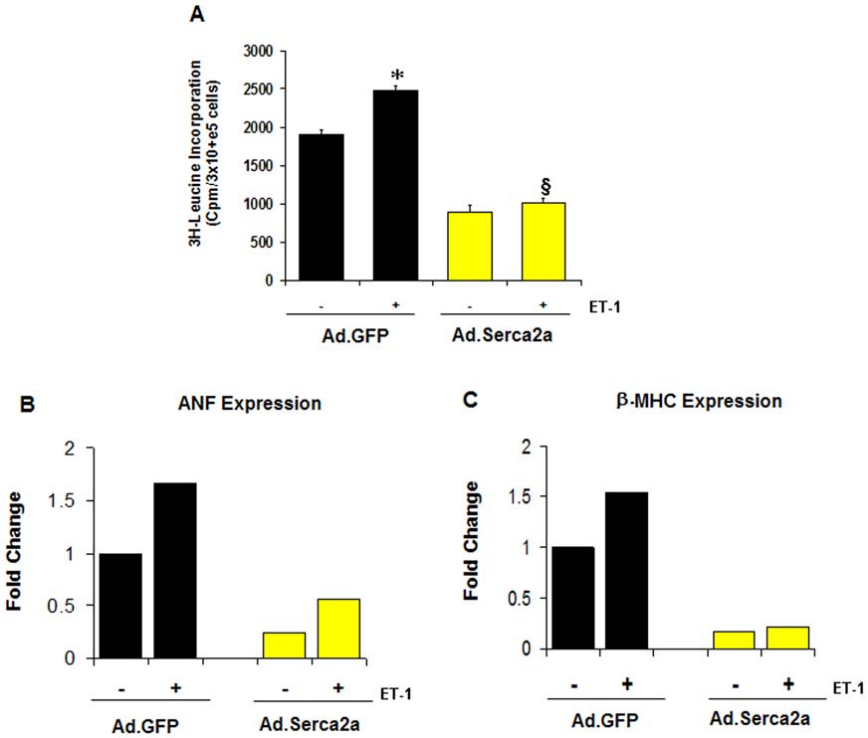
Effects of SERCA2a on phenotypic cardiomyocyte hypertrophy. A) Effect on protein synthesis: The effects of SERCA2a on protein synthesis were evaluated by measuring the rate of ^3^H-Leucine incorporation in neonatal control myocytes infected with Ad.β-gal and myocytes infected with Ad.KChIP2 in the presence (+) or absence (−) of ET-1 stimulation (100 nM). B) Effect of SERCA2a on (B) ANF and (C) β-MHC expression in the presence (+) or absence (−) of ET-1 stimulation (100 nM). SERCA2a overexpression significantly blocked the pronounced increase in ET-1-induced ^3^H-Leucine incorporation, ANF and β-MHC expression.

**Figure 7:**
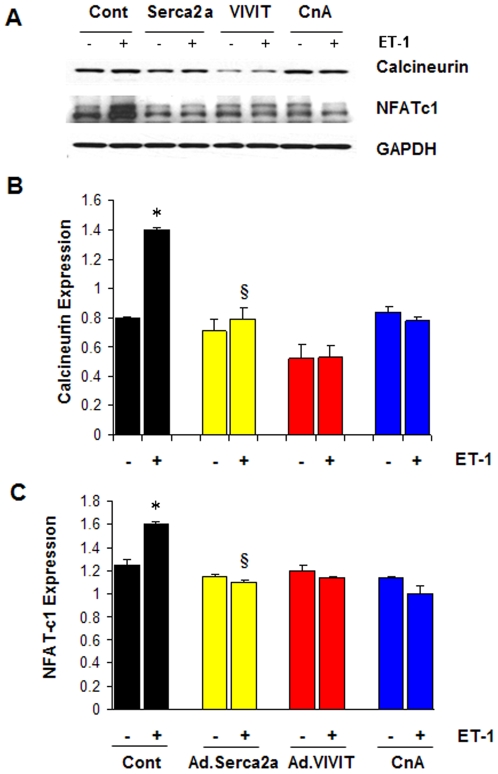
SERCA2a inhibits calcineurin/NFAT pathway. The effect of SERCA2a on calcineurin and NFATc1 expression (A) was evaluated by Western blotting in neonatal myocytes non-infected (Cont) or infected with Ad.SERCA2a in the presence (+) or absence (−) of ET-1. As a comparison, calcineurin and NFATc1 expression was determined in the presence of SERCA2a (Ad.Serca2a) and calcineurin/NFAT-specific inhibitor VIVIT (VIVIT) or Cyclosporin A. Quantitative analysis of mean data (±SEM) of 3 to 4 independent experiments for Calcineurin (B) and NFAT-c1 (C). GAPDH, an internal loading control, is used to normalize protein expression. ^*^ *P*<0.05 Cont+ET-1 vs. Cont; ^§^ *P*<0.05 Ad.Serca2a+ET-1 vs. Cont+ET-1

Hemodynamics measurements (Fig. 1B, C & D) suggest that SERCA2a gene transfer into DM hearts considerably improved myocardial performance as indicated by the reversal of the associated hypertrophy following SERCA2a restoration. We therefore sought to evaluate the effect of SERCA2a on cardiac hypertrophy by investigating whether SERCA2a could modulate any phenotypic changes characteristic of the hypertrophic response, such as enhanced protein synthesis, induction of classical fetal genes (i.e. atrial natriuretic factor (ANF) and β-myosin heavy chain (β-MHC)) and activation of the calcineurin/NFAT pathway [30]. To this end, hypertrophy of neonatal rat cardiac myocytes in culture was obtained after stimulation with endothelin 1 (ET-1). Compared to control, SERCA2a overexpression in ET-1 stimulated myocytes significantly inhibited protein synthesis as measured by [^3^H]-leucine uptake (Fig. 6A) and significantly decreased the mRNA expression of ANF and β-MHC (Fig. 6B & C). We show for the first time that expression of SERCA2a markedly reduced the observed ET-1-induced NFATc1 expression (Fig. 7A). Furthermore, SERCA2a substantially reduced the basal level of NFATc1 expression even in the absence of ET-1 stimulation (Fig. 7A). Likewise, the expression of calcineurin was also significantly decreased by SERCA2a overexpression (Fig. 7A) compared to controls. The abrogation of ET-1-induced NFATc1 and calcineurin by SERCA2a transgene was confirmed by manipulating the expression of these molecules with the highly selective inhibitor of calcineurin/NFAT, VIVIT, and Cyclosporin A (CsA). Similar to SERCA2a, neonatal myocytes incubated with CsA (10 µM) or infected with adenoviral vectors carrying the VIVIT peptide substantially reversed ET-1-induced NFATc1 and calcineurin expression (Fig. 7A). Quantitative analysis of three to four separate determinations is depicted in Fig. 7B (calcineurin) and Fig. 7C (NFAT) normalized to GAPDH. These data indicate that SERCA2a can target the calcineurin/NFAT pathway and may modulate this pathway through its effect on intracellular Ca2 needed for calcineurin activation [30].

## Discussion

The overall goal of this study was to identify the underlying alterations in gene expression associated with cardiomyopathy in type 2 diabetes. The development of diabetic cardiomyopathy and the cellular and molecular perturbations associated with the pathology are complex and multifactorial. These diabetes-related alterations and their cross-interactions can in turn alter signal transduction and gene expression. It is challenging to define the precise factors that regulate gene expression in diabetic cardiomyopathy *in vivo* since the changes observed can be induced by diabetes or diabetes-related abnormalities. In the present study, genomic profiles of heart tissues isolated from non-diabetic rats (LETO) and diabetic rat model of type 2 diabetes (OLETF) were analyzed. In addition, insights into the nature and level of differential myocardial gene expression following normalization of SERCA2a expression in these diabetic hearts were also gained.

### Type 2 diabetes-induced gene expression changes in cardiomyopathy

The data show that diabetes has differentially induced the expression of 838 genes that take part in many cellular processes. Our analysis indicates that diabetic cardiomyopathy is associated with a net downregulation of transcripts (272 up- and 566 down-regulated). The analyses also indicate that diabetic cardiomyopathy in type 2 diabetic rats is markedly associated with changes in genes known to be involved in immune response, development, intracellular signaling, proliferation and transcription. These findings are consistent with known diabetes-induced cellular and molecular cardiac changes, such as structural, metabolic and signaling perturbations [5].

Of particular interest is the observation that diabetes induced high expression levels of resistin-like alpha in the heart. Resistin, a novel hormone that is mainly secreted by adipose tissues in rodents, is thought to be responsible for insulin sensitivity impairment in several rodent models [31]. Recombinant resistin protein was found to impair insulin action in normal mice and cultured adipocytes, and immunoneutralization of resistin improved insulin action in mice with diet-induced obesity [32]. In skeletal muscle cells, resistin was found to regulate the function of IRS-1, Akt1 and GSK-3β and to decrease GLUT4 translocation and glucose uptake in response to insulin [33]. In order to understand what consequences the observed up-regulation of resistin-like alpha may have on the heart, we recently undertook a study in which we showed for the first time that resistin induces cardiac hypertrophy in neonatal cardiomyocytes and contractile abnormalities in adult cardiomyocytes [34]. This observation may be important in providing a basis linking diabetes and hypertrophy and may explain the relevance of the observed high levels of resistin in the diabetic hearts.

### SERCA2a-induced gene expression changes in diabetic cardiomyopathy

We have recently shown that diabetic cardiomyopathic hearts have reduced levels of SERCA2a and that myocardial-targeted expression of SERCA2a in these diabetic hearts improved left ventricular (LV) mechanical and energetics function [22]. Thus, we were particularly interested to discover changes in LV gene expression profile following the rescue of diabetic cardiomyopathy by cardiac overexpression of SERCA2a. Transcriptional analyses showed that SERCA2a gene transfer differentially induced the expression of a subset of genes and appears, in general, to reverse the transcriptional profile induced by diabetes. The transcriptional modulation of many of these genes may have important physiological effects.

#### SERCA2a modulates Cardiac Hypertrophy

The sarcoplasmic reticulum Ca^2+^ ATPase (SERCA2a) plays a pivotal role in intracellular Ca^2+^ handling in cardiomyocytes [35], and its expression is decreased in many models of heart failure including diabetic cardiomyopathy [10], [22], [36]. Hemodynamics measurements (Fig. 1B, C & D) suggest that SERCA2a gene transfer into DM hearts considerably improves myocardial performance as indicated by the reversal of the associated hypertrophy following SERCA2a restoration. To further substantiate this observation we show now that *in vitro* overexpression of SERCA2a abrogates cardiac hypertrophy as evidenced by its significant effect on many phenotypic markers of the hypertrophic response. SERCA2a overexpression in cardiomyocytes remarkably decreased endothelin-1 (ET-1)-induced protein synthesis, ANF and β-MHC expression, as well as calcineurin/NFAT expression. Our *in vitro* data are in close agreement with our recent *in vivo* findings. We showed that in the OLETF type 2 diabetic rat model, transcoronary gene transfer of SERCA2a increased coronary blood flow and lowered the LV weight-to-body weight ratio (LV/BW) mainly by decreasing cardiomyocyte size [37]. Moreover, we showed that an adenoviral short hairpin RNA vector (AdV-shRNA) silenced phospholamban (an endogenous inhibitor of SERCA2a), significantly normalized the massive pressure overload-induced cardiac dilation, and significantly reduced cardiac hypertrophy, cardiomyocyte diameter, and cardiac fibrosis [38]. We also found that SERCA2a overexpression improved anterior wall thickening and reduced ventricular arrhythmias in a rat model of ischemia [20].

Collectively, these data demonstrate that SERCA2a is an important regulator of cardiac hypertrophy. Furthermore, SERCA2-induced regression of myocyte hypertrophy may be mediated, at least, through regulation of the Ca^2+^-regulated phosphatase, calcineurin, which plays a central role in transducing environmental signals that control gene expression and hypertrophic growth in cardiac muscle [30], [39]. Increased SERCA2a activity and/or expression stimulates SR Ca^2+^ uptake thereby diminishing intracellular [Ca^2+^] leading to inactivation of the calcineurin/NFAT signaling pathway.

#### SERCA2a modulation of Ca^2+^ Cycling

In response to SERCA2a normalization, many genes that were either up- or down-regulated were restored back to normal levels and many other genes that were unchanged by the diabetic insult were differentially altered as determined by K-means clustering analysis. The observed differential gene expression following SERCA2a overexpression raises a genuine question as to whether the transcriptional effects of SERCA2a within the cardiomyocyte are direct or occur through SERCA2a's restoration of intracellular Ca^2+^ homeostasis or other factors. Furthermore, SERCA2a overexpression and subsequent enhancement of the sarcoplasmic reticulum (SR) Ca^2+^-ATPase activity can lead to changes in cellular homeostasis and trigger a cascade of molecular cross talk among genes or cellular compartments.

SERCA2a plays an important role in maintaining Ca^2+^ homeostasis in cardiac myocytes. A decrease in SERCA2a levels, as occurs in failing and diabetic hearts, leads to substantial accumulation of diastolic Ca^2+^ which can provide a stimulus for the induction of hypertrophy (and potentially failure) since a variety of kinases, receptor and signaling cascades are directly activated by Ca^2+^ or use Ca^2+^ as a cofactor [30], [40], [41]. Therefore, the restoration of SERCA2a in diabetic hearts has a pleiotropic effect on functional recovery since normalization of Ca^2+^ homeostasis may lead to Ca^2+^-specific gene remodeling. Examples of such an effect are those observed with the down regulation of ryanodine receptor 2 (RyR2), S100A3, calcium binding protein 1 (CaBP1) and the up regulation of calcium channel voltage-dependent L type alpha 1C subunit (Cacna1c), calcium channel voltage-dependent beta 3 subunit (Cacnb3) and calsinelin. The effect of SERCA2a on RyR2 is particularly interesting. Diabetic hearts demonstrate significant decrease in Ca^2+^ transient amplitude and sarcoplasmic reticulum (SR) Ca^2+^ load, and an increase in diastolic Ca^2+^ [42]. This has been primarily attributed to defect in SERCA2a function coupled with abnormal RyR2 activity. Our observation that RyR2 expression is increased in type 2 DM hearts from OLEFT may explain the rise in diastolic Ca^2+^ due to increased Ca^2+^ leaking (release). SERCA2a restoration, however, normalizes this imbalance in Ca^2+^ distribution, possibly through down-regulation of RyR2 expression.

#### SERCA2a modulation of insulin signaling pathway and glucose metabolism

Studies of animal models have demonstrated that insulin signaling pathways are dysregulated in the diabetic myocardium. This is confirmed by our observation that a number of insulin-signaling molecules (PI3K, Akt, Glut4, and atypical PKCλ) were down regulated on the microarray chips in DM hearts. However, SERCA2a gene transfer in these hearts reversed the expression profiles of these molecules. Microarray, real-time PCR and, in some cases, immunoblotting analyses showed increased levels of PI3K, Akt, Glut4 and PKCλ and ndecreased GSK-3β expression following SERCA2a overexpression. SERCA2a's up regulation of Akt in conjunction with GSK-3β down regulation, possibly through Akt signaling, may induce an improvement in ventricular function which is generally observed following SERCA2a overexpression. Furthermore, recent studies have suggested that SERCA2a and the PI3K/GSK3β pathways may interact either directly or through a Ca^2+^-mediated process [43]. GSK-3β was found to negatively regulate the expression of SERCA2a leading to severe systolic and diastolic dysfunction [43], however, treatment with insulin restored SERCA2a to normal levels in diabetic rats [10] and inhibited the activity of GSK-3β through increased phosphorylation of Akt [44]. The interrelationship of the effects of SERCA2a and insulin pathway appear to be beneficial to the myocardium in pathological conditions such as diabetes.

Increasing evidence suggests that impaired cardiac energetics contribute to the contractile dysfunction of the cardiomyopathic heart. A key element in this process is the perturbation of the glucose metabolism which is partially due to dysregulated insulin signaling. We recently demonstrated that SERCA2a overexpression in DM hearts improved myocardial bioenergetics [22]. This is supported by our current findings that SERCA2a up-regulates the expression of many transcripts involved in energy metabolism, for instance creatine kinase, and glucose transport, such as PKCλ, Akt, operating downstream of PI3K, and Glut4, which are known to play critical roles in insulin- stimulated glucose transport and regulation. In addition to Glut-4-mediated glucose uptake, stimulation of glycolysis can also be enhanced by increasing levels of fructose-2-6-phosphate through activation of 6-phosphofructo-2-kinase (PFK-2) [45] and pyruvate dehydrogenase (PDH) through activation of pyruvate dehydrogenase phosphate phosphatase (PDHP) [46]. Our microarray data show that SERCA2a has increased the expression of PFK-2 (1.83 fold) and PDHP (1.7 fold), as well as glucose phosphate isomerase (1.6 fold), which may translate into an enhancement of glucose metabolism in the diabetic heart. These findings are in general agreement with recent findings that SERCA2a overexpression in transgenic mice results in increased glucose oxidation [47]. Increasing SERCA2a expression leading to an increase in Ca^2+^ maintenance by the SR may lead to an effective energy metabolism and subsequently an improvement in cardiac performance. In fact, ameliorating calcium homeostasis in failing [26] as well as diabetic hearts [22] results in improved cardiac metabolism and energetics. Reciprocally, improving myocardial energetics in hypertrophic [48] and failing hearts [49] normalized calcium handling.

In summary, we have explored the pattern of gene expression changes that occur in diabetic heart failure before and after restoration of the intracellular Ca**^2+^** homeostasis and contractile function using *in vivo* adenoviral gene transfer of SERCA2a. We have observed that SERCA2a restoration has affected many genes that are involved in multiple cellular processes. It is very likely that some of these processes are directly mediated by Ca^2+^ and others are the results of a reprogramming response to intracellular cross talks among genes or cellular compartments as a result of better Ca^2+^ signaling and sustained changes in sarcoplasmic reticulum Ca^2+^ contents. The differential regulation of these genes may shed light on the role of SERCA2a and the beneficial effects which accompany its restoration in diabetic failing hearts.

### Limitations

Although the analysis of gene expression using microarray technology is a powerful technique to improve disease understanding, it does have its limitations which are related to tissue selection, the lack of a unified approach to chip data analysis, the presentation of vast amounts of results, and the extrapolation and generalization of the findings [50]. In addition, the outcome of microarray analysis is sensitive to the design and the number of probes on the array. We used a microarray chip with approximately 22,000 printed probes; however, these are not all rat genes currently known, and therefore, the knowledge obtained from the present experiments remains incomplete. Another limitation is represented by the data validation. The vast amount of genes obtained is by and large more than the validation of the data, which is limited to selected genes of specific interest.

Another limitation is that our findings represent a snapshot examination of gene transcriptional profile events in the progression of diabetic cardiomyopathy. However, valuable information can still be obtained on the molecular fingerprinting and the potential dysregulated pathways associated with diabetes-induced cardiac dysfunction. Confirming these molecular changes is essential before any definitive conclusion can be drawn to correlate these changes with the disease. We have chosen a time point after which the OLETF diabetic rats have an overt diastolic and systolic dysfunction with a clear down-regulation of the Ca^2+^ ATPase SERCA2a. Restoring SERCA2a at this time point and evaluating the targeted transcriptome would be more insightful in understanding its biological effects. The differential transcriptional changes produced by diabetes and SERCA2a suggest that the two conditions have different remodeling effects on the myocardium. However, it would also be interesting to determine the molecular alterations before and after the onset of systolic and diastolic dysfunction associated with diabetic cardiomyopathy. Finally, our study examined transcription changes in whole hearts; thus further studies are warranted to define the contribution of individual cardiomyocytes or fibroblasts to the effect of diabetes on cardiac function.
